# Effect of the Japanese Herbal Kampo Medicine Dai-Kenchu-To on Postoperative Adhesive Small Bowel Obstruction Requiring Long-Tube Decompression: A Propensity Score Analysis

**DOI:** 10.1155/2011/264289

**Published:** 2011-03-31

**Authors:** Hideo Yasunaga, Hiroaki Miyata, Hiromasa Horiguchi, Kazuaki Kuwabara, Hideki Hashimoto, Shinya Matsuda

**Affiliations:** ^1^Department of Health Management and Policy, Graduate School of Medicine, The University of Tokyo 113-0033, 7-3-1, Hongo, Bunkyo-ku, Tokyo, Japan; ^2^Department of Health Quality Assessment, Graduate School of Medicine, The University of Tokyo 113-0033, 7-3-1, Hongo, Bunkyo-ku, Tokyo, Japan; ^3^Department of Health Care Administration and Management, Graduate School of Medical Sciences, Kyushu University, 3-1-1 Maidashi, Higashi-ku, Fukuoka 812-8582, Japan; ^4^Department of Health Economics and Epidemiology Research, School of Public Health, The University of Tokyo 113-0033, 7-3-1, Hongo, Bunkyo-ku, Tokyo, Japan; ^5^Department of Preventive Medicine and Community Health, University of Occupational and Environmental Health, 1-1, Iseigaika, Yahatanishi-ku, Kitakyushu, Fukuoka 807-8555, Japan

## Abstract

Adhesive small bowel obstruction (ASBO) is an adverse consequence of abdominal surgery. Although the Kampo medicine Dai-kenchu-to is widely used in Japan for treatment of postoperative ASBO, rigorous clinical studies for its use have not been performed. In the present retrospective observational study using the Japanese diagnosis procedure combination inpatient database, we selected 288 propensity-score-matched patients with early postoperative ASBO following colorectal cancer surgery, who received long-tube decompression (LTD) with or without Dai-kenchu-to administration. The success rates of LTD were not significantly different between Dai-kenchu-to users and nonusers (84.7% versus 78.5%; *P* = .224), while Dai-kenchu-to users showed a shorter duration of LTD (8 versus 10 days; *P* = .012), shorter duration between long-tube insertion and discharge (23 versus 25 days; *P* = .018), and lower hospital charges ($23,086 versus $26,950; *P* = .018) compared with Dai-kenchu-to nonusers. In conclusion, the present study suggests that Dai-kenchu-to is effective for reducing the duration of LTD and saving costs.

## 1. Introduction

Adhesive small bowel obstruction (ASBO) is an adverse consequence of abdominal surgery, which can result in overall prolonged recovery coupled with augmented patient discomfort and an increased risk of postoperative complications [1, 2]. Approximately 3% of all surgical admissions are attributable to intestinal adhesions following abdominal surgeries [3]. In particular, colorectal cancer surgery is considered a high risk of postoperative ASBO. A previous report showed that intestinal obstruction due to adhesion occurs in approximately 11% of patients who undergo total colectomy [3].

Long-tube decompression (LTD) has been shown to be successful for patients with clinically and radiographically diagnosed ASBO, except for cases with contraindications including strangulation, malignant obstruction, peritonitis, and radiation enteritis [2, 4, 5]. A long tube is inserted through the nose, esophagus, stomach, and duodenum. A balloon is then inflated, and peristalsis advances the tube to the point of obstruction, decompressing the bowel of air and fluids. In Japan, Dai-kenchu-to, which is a Kampo medicine (traditional Japanese herbal medicine), is used for prevention and treatment of postoperative ASBO [6, 7]. Dai-kenchu-to products are made by two different Kampo pharmaceutical companies (Tsumura and Kotaro). Both products comprise the same ingredients as follows: extract granules of 5.0 g processed ginger (*kankyo*), 3.0 g ginseng (*ninjin*), and 2.0 g zanthoxylum fruit (*sansho*) in the daily dosage.

Several pharmacological mechanisms of Dai-kenchu-to have been confirmed mainly based on animal experiments, including an increase in blood flow in the intestinal tract [8], direct stimulation of intestinal motility [9–12], and prevention of bacterial translocation [13]. Previous studies have also confirmed that Dai-kenchu-to elevates levels of neurotransmitters or hormones in human plasma, including motilin, vasoactive intestinal polypeptide, calcitonin gene-related peptide, serotonin, and substance P [14–17]. Several clinical studies have suggested an effect of Dai-kenchu-to on postoperative ASBO patients. Orally administered Dai-kenchu-to reduces postoperative ASBO-related symptoms [6], the frequency of secondary laparotomy [18], and length of hospital stay [19]. However, these previous studies were based on a small number of patients. For patients with severe postoperative ASBO requiring LTD, direct administration of Dai-kenchu-to into the small intestine through a long tube is often attempted in the Japanese clinical setting although the effect of such a method remains unclear [12, 20]. While Dai-kenchu-to is widely used in Japan, rigorous clinical studies are lacking and there is a paucity of data for assisting physicians in other countries to make a decision on trying this type of medicine for postoperative ASBO [6].

The purpose of this study was to investigate the effectiveness of transluminal Dai-kenchu-to administration for patients with postoperative ASBO requiring LTD after colorectal cancer surgery, using the diagnosis procedure combination (DPC) inpatient database in Japan. We hypothesized that transluminally administered Dai-kenchu-to is effective for reducing a secondary operation for removal of intra-abdominal adhesion, duration of LTD, length of stay and hospital charges.

## 2. Methods

### 2.1. Data Source

DPC is a Japanese case-mix classification system that is similar to the diagnosis-related groups in the US medicare system. This patient classification system was launched in 2002 by the Ministry of Health, Labour, and Welfare of Japan, and was linked with a lump-sum payment system [21]. All of Japan's 82 teaching hospitals are obliged to adopt the DPC system, but community hospitals can voluntarily adopt it. A survey of DPC hospitals is conducted between July 1 and December 31 every year by the DPC Research Group funded by the Ministry of Health, Labour, and Welfare of Japan [22, 23]. Not only administrative claims data but also detailed patient data are collected for all inpatients discharged from participating hospitals between July 1 and December 31. The survey began in 2003 with 82 teaching hospitals and the number of participating hospitals has steadily increased, with 926 in 2007 and 855 in 2008. The number of inpatients was 2.99 million in 2007 and 2.86 million in 2008. The number of inpatients in 2008 (2.86 million) represented approximately 40% of all inpatient admissions to acute care hospitals in Japan.

The DPC database includes the following data: patients' age and sex; main diagnoses, comorbidities at admission and complications after admission recorded with the international classification of diseases, tenth revision codes and text data in the Japanese language, procedures coded with the Japanese original codes, drugs and devices used, length of stay (days), and in-hospital mortality.

The DPC database partially corresponds to the nationwide inpatient sample in the United States [24], but it has several advantages. In the DPC database, complications that occur after admission are clearly differentiated from comorbidities that were already presented at admission. For example, we were able to separate ASBO that developed prior to hospital admission from ASBO that developed during hospitalization. For optimizing the accuracy of the recorded diagnoses, physicians in charge are obliged to record the diagnoses with reference to medical charts. The dates when patients are admitted or discharged, and when each procedure is performed, they are recorded using a uniform data submission format. For example, the dates when the long tube is inserted and removed and the dates when administration of Dai-kenchu-to is started and terminated are recorded. 

All patient identifiers were removed from this database. The requirement for informed consent was waived because of the anonymous nature of the data. Study approval was obtained from the institutional review board at the University of Occupational and Environmental Health.

### 2.2. Patient Selection

First, among all the 5.9 million inpatients between July 1 and December 31 in 2007 and 2008, we selected patients who underwent radical surgery for colorectal cancer including colectomy, high anterior resection of the rectum, low anterior resection of the rectum, and abdominoperineal resection. Surgical approaches (open or laparoscopic) were also identified. Second, we excluded patients with a diagnosis of strangulation obstruction, malignant obstruction, peritonitis, or radiation enteritis. We also excluded patients with inflammatory bowel disease (Crohn's disease or ulcerative colitis) because it has an intensive risk of intra-abdominal adhesion [4]. Third, we identified patients who started LTD after the date of colorectal cancer surgery and selected the following two groups of patients: (1) patients who used Dai-kenchu-to during the period of LTD, that is, patients in whom Dai-kenchu-to was transluminally administered though a long tube (Dai-kenchu-to users) and (2) patients who did not use Dai-kenchu-to during the whole period of hospitalization (Dai-kenchu-to nonusers).

### 2.3. Patient Background

Patient background data that could potentially affect the endpoints were assessed including age, sex, comorbidities, and type of surgery performed. Comorbidities assessed included hypertension, diabetes, chronic heart disease (ischemic heart disease, valvular heart disease, cardiomyopathy, or congenital heart disease), history of cerebrovascular diseases (cerebral infarction, cerebral hemorrhage, or subarachnoid hemorrhage), chronic lung disease (emphysema, chronic bronchitis, bronchiectasis, asthma, interstitial lung disease, or pulmonary hypertension), chronic renal failure, and liver cirrhosis. We also assessed hospital volume for colorectal cancer surgery because it could potentially affect postoperative clinical consequences including mortality and length of stay [25]. Hospital volume was determined as the number of all the radical surgeries for colorectal cancer during the study period, using the unique identifier for each hospital.

### 2.4. Endpoints

“Successful” LTD cases were defined as those in whom a long tube was finally removed and who were discharged home or to a rehabilitation hospital. “Unsuccessful” LTD cases were defined as those who required secondary laparotomy for removal of adhesion or those who died during the hospitalization. Two secondary endpoints were determined as follows: (i) duration of LTD (duration between long-tube insertion and removal) and (ii) duration between long-tube insertion and discharge. Another surrogate endpoint was total hospital charges. The exchange rate was assumed to be JP ¥100 = US $1.

### 2.5. Statistical Analyses

We used propensity score matching [26] to adjust for differences in baseline characteristics, because the patients were not randomly assigned to receive Dai-kenchu-to. We performed a one-to-one matched analysis on the basis of estimated propensity scores of each patient. The log odds of the probability that a patient received Dai-kenchu-to was modeled for potential confounders including age, sex, comorbidities, type of surgery, and hospital volume. C-statistics were calculated for evaluating the goodness of fit. The estimated propensity scores were compared between Dai-kenchu-to users and nonusers, and a “match” occurred when one patient in the Dai-kenchu-to user group had an estimated score within 0.6 standard deviation of another patient in the Dai-kenchu-to nonuser group. If two or more patients in the Dai-kenchu-to user group met this criterion, we randomly selected one patient for matching.

We performed univariate comparisons of patient characteristics and outcome variables between the propensity-score-matched groups of Dai-kenchu-to users and non-users, using Fisher's exact tests and *t*-tests as appropriate. A logistic regression analysis was performed where the independent variable was determined as successful LTD (successful LTD = 1; unsuccessful LTD = 0). We compared (i) the rates of patients who terminated LTD and (ii) the rates of patients who were discharged between Dai-kenchu-to users and nonusers, using Kaplan-Meier methods and log-rank tests. Cox regression analyses were performed to model the concurrent effect of various factors on (i) termination of LTD and (ii) discharge. A *P* value <.05 was considered statistically significant. All statistical analyses were conducted using PASW version 18.0 (SPSS Inc.; Chicago, IL, US).

## 3. Results

We identified a total of 603 eligible patients who underwent LTD after colorectal cancer surgery, including Dai-kenchu-to users (*n* = 453) and Dai-kenchu-to nonusers (*n* = 150). Using one-to-one propensity score matching, we selected 144 couples of Dai-kenchu-to users and nonusers. C-statistics showed that the goodness of fit was 0.592 in this model. 

Table 1 shows the patients' backgrounds in 288 selected cases of Dai-kenchu-to users and non-users. There were no significant differences in the patients' background between Dai-kenchu-to users and non-users. No Kampo formulation other than Dai-kenchu-to was administered to any of the 288 investigated patients during hospitalization.

LTD was successful in 113 (78.5%) of 144 Dai-kenchu-to nonusers and 122 (84.7%) of 144 Dai-kenchu-to users. A secondary operation was performed in 48 (16.7%) of the 288 patients, including 28 (19.4%) in Dai-kenchu-to nonusers and 20 (13.9%) in Dai-kenchu-to users. In-hospital deaths were identified in four (2.8%) of the Dai-kenchu-to nonusers and two (1.4%) of the Dai-kenchu-to users. Fisher's exact tests showed no significant differences in the success rate of LTD (*P* = .224), the rate of secondary operation (*P* = .206) and in-hospital mortality (*P* = .684) between the two groups.  Table 2 shows the results of logistic regression analysis for successful LTD. The occurrence of successful LTD was not significantly different between Dai-kenchu-to users and nonusers (odds ratio, 1.60; 95% confidence interval (CI), 0.86–2.95; *P* = .137). 

**Figure 1:**
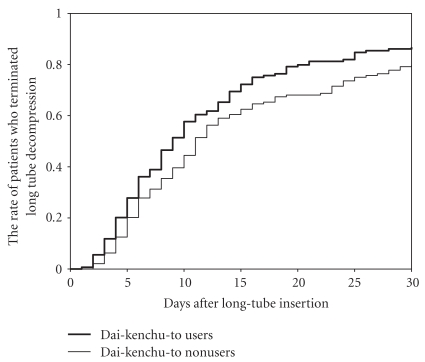
Days after long-tube insertion and the rate of patients who terminated long-tube decompression (LTD) in Dai-kenchu-to (DKT) users and nonusers.

**Figure 2:**
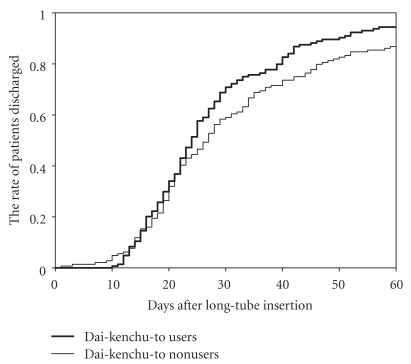
Days after long-tube insertion and the rate of patients who were discharged in Dai-kenchu-to (DKT) users and nonusers.

The median and interquartile range (IQR) of duration of LTD was 10 days (6–17 days) in Dai-kenchu-to non-users and 8 (5–15) days in Dai-kenchu-to users. Figure 1 shows the rate of patients who terminated LTD in relation to days after long-tube insertion. A log-rank test showed a significant difference between the groups (*P* = .012). The median (IQR) of duration between long-tube insertion and discharge was longer in Dai-kenchu-to nonusers (25 (19–36) days) compared with that in Dai-kenchu-to users (23 (18–31) days).  Figure 2 shows the rate of patients who were discharged after long tube insertion. A log-rank test showed a significant difference between the groups (*P* = .018).  Table 3 shows the results of the Cox regression analyses. After adjusting for the patient background, the Dai-kenchu-to user group showed a significantly earlier termination of LTD (hazard ratio, 1.41; 95% CI, 1.08–1.84; *P* = .011) compared with the Dai-kenchu-to non-user group; therefore, the duration of LTD was significantly shorter in Dai-kenchu-to users than that in nonusers. The Dai-kenchu-to user group showed a significantly earlier discharge after long tube insertion (hazard ratio, 1.40; 95% CI, 1.08–1.83; *P* = .012) compared with the Dai-kenchu-to nonuser group; therefore, the duration between long-tube insertion and discharge was significantly shorter in Dai-kenchu-to users than that in nonusers. 

The mean (±SD) total hospital charges were significantly lower in Dai-kenchu-to users (23,086 ± 9,427 US dollars) than those in Dai-kenchu-to nonusers (26,950 ± 17,025 US dollars; *P* = .018).

## 4. Discussion

Laparotomy and intestinal manipulation may cause decreased gastrointestinal motility, delayed gastrointestinal transit, and intra-abdominal adhesion [1]. Adhesion may cause mechanical small bowel obstruction, resulting in prolonged hospitalization and greater health care utilization [27]. In the Japanese clinical setting, Dai-kenchu-to is commonly used for prevention and treatment of postoperative ASBO [6, 7]. Dai-kenchu-to is considered a safe drug and only a rare incidence of minor side effects has been reported [6, 18]. The Japanese government has covered Dai-kenchu-to as a prescription drug under its national health insurance since 1986, and currently, approximately 500 million Dai-kenchu-to sachets are prescribed annually in Japan [6].

Few rigorous clinical studies have been performed to investigate the effects of Kampo regimens against any specific disease entities [28–30]. Dai-kenchu-to has been shown to have several pharmacological effects using animal experiments [8–13]. Limited data from several previous clinical studies have suggested the effectiveness of Dai-kenchu-to in the prevention of or early recovery from postoperative ASBO [18–20]. In a previous randomized study of 24 patients with postoperative ASBO, the frequency of a secondary operation was significantly lower in patients who received Dai-kenchu-to compared with placebo [18]. In a retrospective study of 69 patients with postoperative ASBO, the duration of LTD and average length of stay were significantly shorter in patients who transluminally received Dai-kenchu-to than those in patients who did not [20]. However, these studies were based on a small number of patients. The present larger-scale study demonstrated that administration of Dai-kenchu-to through a long tube significantly reduced the duration of LTD and hospital stay after long-tube insertion. Furthermore, the use of Dai-kenchu-to significantly reduced total hospital charges. The success rate of LTD in Dai-kenchu-to users (84.7%) appeared to be higher than that in nonusers (78.5%), but this difference was not significant (*P* = .224). Although the sample size in our study was much larger than that in previous studies, our study still may have been underpowered for identifying a significant reduction in secondary surgery or in-hospital deaths in Dai-kenchu-to users. Therefore, further study with more samples is required.

Several limitations to the study should be acknowledged. Although we used propensity score matching to adjust for differences in baseline characteristics, this study was based on a nonrandomized, retrospective study. We had no data on obesity, cancer stage, or past history of laparotomies, which may have affected the difficulty of surgical procedures. The database also lacked information on some factors that could potentially affect postoperative consequences, such as smoking [31].

## 5. Conclusion

Our propensity-score-matched analysis suggests that Dai-kenchu-to is effective in terms of reducing the duration of LTD and saving costs for patients with ASBO after colorectal cancer surgery. Further study with larger samples or a randomized controlled trial is necessary for obtaining more accurate data on this medicine.

## Figures and Tables

**Table 1:** Patient background.

	Total		Dai-kenchu-to nonusers		Dai-kenchu-to users		*P*
	(*N* = 288)		(*N* = 144)		(*N* = 144)		
Age (average ± SD)	68.2 ± 9.6		68.4 ± 10.1		67.9 ± 9.1		.677
Sex (male) (*n*, %)	213	74.0	110	76.4	103	71.5	.347
Comorbidities (*n*, %)							
Hypertension	53	18.4	28	19.4	25	17.4	.648
Diabetes	48	16.7	24	16.7	24	16.7	1.000
Cardiovascular diseases	16	5.6	8	5.6	8	5.6	1.000
Chronic lung diseases	6	2.1	3	2.1	3	2.1	1.000
Cerebrovascular diseases	2	0.7	1	0.7	1	0.7	1.000
Chronic renal failure	2	0.7	0	0.0	2	1.4	.156
Liver cirrhosis	1	0.3	0	0.0	1	0.7	.316
Type of surgery (*n*, %)							
Colectomy	144	50.0	71	49.3	73	50.7	
High anterior resection of rectum	24	8.3	12	8.3	12	8.3	.962
Low anterior resection of rectum	70	24.3	35	24.3	35	24.3	
Abdominoperineal resection	50	17.4	26	18.1	24	16.7	
Approach							
Laparoscopic	21	7.3	11	7.6	10	6.9	.821
Open	267	92.7	133	92.4	134	93.1	
Hospital volume for colorectal surgery (per month; average ± SD)	8.3 ± 5.9		8.2 ± 5.8		8.3 ± 6.1		.879

**Table 2:** Logistic regression analysis for successful long-tube decompression.

	95% confidence		
	Odds ratio	Interval	*P*
Dai-kenchu-to			
Nonusers	Reference		
Users	1.60	0.86–2.95	.137
Age (10-yr age increase)	1.09	0.79–1.51	.592
Sex (Female)	0.52	0.27–1.01	.054
Diabetes	0.73	0.33–1.60	.428
Hypertension	1.07	0.47–2.42	.873
Cardiac diseases	0.45	0.15–1.40	.169
Chronic lung diseases	0.84	0.09–7.74	.879
Hospital volume (per month)	0.99	0.94–1.05	.820
Type of surgery			
Colectomy	Reference		
Rectal of the resection	0.95	0.47–1.94	.893
Abdominoperineal resection	0.62	0.28–1.41	.254
Open or laparoscopic surgery			
Open	Reference		
Laparoscopic	0.85	0.26–2.72	.779

**Table 3:** Cox regression analyses for termination of long-tube decompression and discharge.

	Termination of long-tube decompression			Discharge		
	95% confidence			95% confidence		
	Hazard ratio	Interval	*P*	Hazard ratio	Interval	*P*
Dai-kenchu-to						
Nonusers	Reference			Reference		
Users	1.41	1.08–1.84	.011	1.40	1.08–1.83	.012
Age (10-yr age increase)	0.99	0.86–1.15	.910	0.90	0.78–1.04	.147
Sex (female)	0.79	0.58–1.09	.150	0.74	0.54–1.01	.058
Diabetes	0.93	0.64–1.35	.701	0.78	0.53–1.13	.186
Hypertension	1.23	0.87–1.74	.248	1.45	1.02–2.04	.036
Cardiac diseases	0.53	0.28–1.03	.060	0.46	0.24–0.87	.016
Cerebrovascular diseases	1.26	0.29–5.42	.757	0.40	0.10–1.68	.211
Chronic lung diseases	0.50	0.20–1.22	.128	0.51	0.21–1.27	.148
Hospital volume per month	1.00	0.98–1.02	.973	1.02	1.00–1.04	.076
Type of surgery						
Colectomy	Reference			Reference		
Rectal resection	0.69	0.51–0.94	.018	0.78	0.58–1.05	.097
Abdominoperineal resection	0.72	0.49–1.06	.093	0.71	0.49–1.04	.081
Open or laparoscopic surgery						
Open	Reference			Reference		
Laparoscopic	1.27	0.73 – 2.23	.398	1.37	0.82–2.27	.228
